# The impact of smartphone addiction and negative emotions on parent–child relationships among elementary school students

**DOI:** 10.3389/fpsyt.2025.1582741

**Published:** 2025-06-02

**Authors:** Ze Geng, Ran Liu

**Affiliations:** ^1^ Institute for High-quality Development Strategies of Education in Ethnic Minority Areas, Minzu University of China, Beijing, China; ^2^ School of Education, Minzu University of China, Beijing, China

**Keywords:** smartphone addiction, negative emotions, emotional regulation, parent-child relationships, primary school students, structural equation modeling

## Abstract

**Background:**

The widespread prevalence of smartphones has profoundly influenced the lives of individuals across all age groups, with children being particularly affected. The issue of smartphone addiction among primary school students has emerged as a global concern as it gives rise to a multitude of adverse outcomes, including depression, anxiety, and strained parent–child relationships.

**Aims:**

This study investigated correlations among smartphone addiction (encompassing four dimensions: loss of control, withdrawal, escapism, and inefficiency), negative emotions, and parent-child relationships, while exploring how these factors interact.

**Methods:**

Data were collected from 1,144 Chinese primary school students (aged 6–15) via parent-completed questionnaires: the Mobile Phone Addiction Index (MPAI), Emotion Questionnaire, and Child-Parent Relationship Scale (CPRS). Structural equation modeling (SEM) analyzed relationships among variables.

**Results:**

1. Smartphone addiction dimensions divergently impacted emotions: Withdrawal and inefficiency exacerbated negative emotions and emotional regulation difficulties, while loss of control and escapism partially alleviated them—a novel finding challenging traditional unidimensional addiction frameworks. 2. Differential effects of smartphone addiction on parent - child relationships: Loss of control correlated with positive parent-child intimacy; withdrawal increased conflict and dependence; escapism reduced conflict and dependence; inefficiency led to high conflict, high dependence, and low intimacy. 3. The impact of emotions caused by smartphone addiction on parent - child relationships: Negative emotions and emotional regulation difficulties significantly worsened parent-child relationships, manifesting as reduced intimacy, increased conflict, and heightened dependence.

**Conclusion:**

This study explored the relationships among smartphone addiction, negative emotions, and parent-child relationships in primary school students. Different dimensions of addiction have different impacts on negative emotions and emotional regulation. Both addiction and negative emotions significantly affect parent - child relationships. The research results are of great significance to researchers, educators, and parents, and can help promote children’s healthy growth and harmonious parent - child relationships.

## 1 Introduction

Widespread use of smartphones has profoundly changed the lives of people across all age groups. According to the 55th Statistical Report on the Development of the Internet in China, published by the China Internet Network Information Center (CNNIC) in 2025, the number of mobile internet users in China had reached 1.105 billion by December 2024. Since December 2023, the number of users has grown by 14.03 million. Currently, 99.7% of internet users utilize mobile phones to access the internet. Among them, teenagers accounted for 49% of new internet users (1).

As digital natives, teenagers have become accustomed to using smartphones for learning, entertainment, and gaming (2). Smartphone addiction is an emerging behavioral addiction that has been attracting increasing research attention. This addiction is characterized by symptoms such as a subjective sense of loss of control and withdrawal reactions due to excessive smartphone use (3, 4), and it has become a global issue (5). Numerous studies have demonstrated that excessive smartphone use can lead to a range of negative outcomes, such as poor sleep quality (6), headaches, indigestion, and blurred vision (7), as well as psychological problems, such as depression, anxiety (8), low self-esteem (9), lack of self-control (10), and psychological distress (11, 12). Additionally, smartphone addiction has triggered many social issues, such as a decline in academic performance (11, 13), strained interpersonal relationships (14, 15), and increased family conflicts (16). Elementary school-aged children are transitioning from childhood to adolescence (17). Many children receive their first smartphone during this period, and their ability for self-control is still immature. Children with a high susceptibility to smartphone addiction are prone to persistently encounter addiction–related problems in both adolescence and adulthood (18). Therefore, it is particularly important to intervene in children’s smartphone use during elementary school.

According to ecological systems theory (19), to understand teenagers’ behavior patterns, it is necessary to consider their growth environment as a whole. Problematic behavior in adolescents is often closely related to their family environment, especially their relationship with their parents (20, 21) and their parents’ parenting style (22). The parent–child relationship is one of the most important interpersonal relationships that exists during elementary school students’ development, playing a crucial role in their physical and mental health (23). According to attachment theory, a good parent–child relationship provides emotional support, security, and proper value guidance, helping children form a positive self-concept and healthy personality (24). However, when adolescents face difficulties in establishing intimate relationships with their parents, they may seek emotional compensation through connections with material objects or behaviors (25). Smartphones are important tools for maintaining relationships and are more likely to become attachment objects for emotional compensation (26). Similarly, smartphone addiction can interfere with the normal development of parent–child relationships. On one hand, excessive smartphone use by children reduces face-to-face communication and interaction with their parents, weakening emotional bonds (27); on the other hand, conflicts and contradictions arising from smartphone use can increase tension in the parent–child relationship (28). Therefore, smartphone addiction and parent–child relationship influence each other. However, existing research has focused more on exploring how parent–child relationships affect smartphone addiction (29–31), with less attention paid to exploring the impact of smartphone addiction on various aspects of the parent-child relationship. On the basis of this, we put forward the Hypothesis 1 (H1): Smartphone addiction has a significant impact on parent–child relationships.

Negative emotions refer to emotional states that arise when people express negative feelings toward specific events or individuals (32). The degree of smartphone addiction is closely related to negative emotional states, and the risk of addiction increases with the exacerbation of negative emotions, suggesting a potential reciprocal influence (33). From a theoretical perspective, smartphone addiction can lead to negative emotions, such as depression, anxiety, and loneliness (6, 14), as excessive smartphone use disrupts normal life rhythms and social interactions, affecting sleep quality (8). Additionally, according to compensatory Internet use theory (34), individuals may turn to smartphones to alleviate negative emotions, seek comfort in the virtual world, and escape real-life problems (35), thereby forming a vicious cycle. This relationship is influenced by individual factors (such as self-control and personality traits) (4, 36), family factors (parent–child relationship quality, parenting styles) (20, 21), and social factors (social support and cultural environment) (37).

Students in different educational stages face diverse stressors, and the moderating role of smartphone addiction between stressors and negative emotions also varies. During the vocational training stage, students are confronted with the dual pressures of theoretical learning and practical internships. Some scholars’ research has found that challenge stressors are negatively correlated with smartphone addiction and can help students cope with stress, while obstacle stressors, through smartphone addiction, indirectly increase cyberloafing behavior (38). This makes students with a high degree of addiction more likely to avoid stress and exacerbate negative emotions, while those with a low degree of addiction are better at actively coping. In the university stage, students face numerous pressures such as social interaction, academics, and career planning. Smartphone addiction may lead them to engage in phubbing behavior, ignore real - world challenges, cause self - cognitive biases, increase negative emotions, and have an adverse impact on social and mental health (39).

Difficulties in emotional regulation also increase the risk of smartphone addiction (40). Some studies have pointed out that adolescents addicted to the Internet face more challenges in emotional regulation, particularly in terms of excessive suppression of emotional expression and infrequent use of cognitive reappraisal strategies (41, 42). When faced with negative emotions, individuals with emotional regulation difficulties are less able to regulate their emotional state on their own and are more likely to rely on external means to soothe their emotions (43). Over - suppressing negative emotional experiences might exacerbate the link between negative emotions and Internet addiction. As a result, challenges in emotion regulation could further reinforce the relationship between negative emotions and smartphone addiction (44). According to cognitive behavioral theory, smartphone addiction can also interfere with normal emotional regulation strategies (45). Addicts tend to display impulsive personality traits when faced with negative emotions, seeking temporary relief through their phones (46) instead of using effective emotional regulation methods to handle their emotions, thereby further leading to difficulties in emotional regulation. Based on this, we proposed Hypothesis 2 (H2): Smartphone addiction significantly affects children’s negative emotions and difficulties in emotional regulation.

Current research on parent–child relationships and negative emotions mainly focuses on how parent–child relationships influence children’s negative emotions, emphasizing that individuals with good parent–child relationships show better social skills and fewer negative emotional expressions than those with poor relationships (47). In a positive parent–child relationship, children can feel their parents’ love, support, and understanding, which creates a safe environment for learning emotional regulation and provides a good model. Through positive guidance and demonstration, parents help children identify and understand their emotions, teach them effective emotional regulation methods, and promote emotional regulation development (48). In contrast, poor parent–child relationships, such as those with frequent conflicts or emotional distance, deprive children of the guidance and support needed for emotional regulation, making it more difficult for them to cope effectively with negative emotions and increasing the risk of falling into a negative emotional cycle (49).

In fact, negative emotions also have a significant impact on parent–child relationships. For children, prolonged negative emotional states, such as anxiety and depression, may manifest as behavioral issues, such as irritability and rebellion, which can lead to parent–child conflicts and reduce intimacy (14). Moreover, from the parents’ perspective, negative emotions can affect parenting styles. For example, parents who are under pressure at work or in a bad mood may lack patience with their children, adopting authoritarian or neglectful parenting styles, which not only harms the family atmosphere but also makes children feel emotionally neglected, thus impacting the quality of the parent–child relationship (50). Similarly, children with good emotional regulation can better cope with negative emotions and avoid conflicts with parents due to emotional outbursts, which helps maintain a good parent–child relationship (43). Therefore, we proposed Hypothesis 3 (H3): Children’s negative emotions and emotional regulation difficulties significantly impact parent–child relationships.

In 2008, Leung categorized smartphone addiction into four types: uncontrollability (spending excessive time on a smartphone without control); withdrawal (experiencing frustration when unable to use the phone normally); escapism (using the phone to escape loneliness, anxiety, and other real-life problems); and inefficiency (excessive phone use affecting daily life and study efficiency) (51). Pianta classified parent–child relationships into intimacy (deep and warm emotional connections between parents and children), conflict (ongoing conflicts between parents and children), and dependency (children displaying dependence on parents) (52).

To further analyze the effects of smartphone addiction, negative emotions, and parent–child relationships, this study will design the following sub-hypotheses based on the nine extracted factor variables(“uncontrollability, withdrawal, escapism, inefficiency, negative emotions, emotional regulation difficulties, intimacy, conflict, and dependency.”) (**Table 1**).

**Table 1:** Main Hypotheses and Sub-Hypotheses Statistics Table.

Main Hypotheses	Sub-Hypotheses
**Hypothesis 1 (H1)**: Smartphone addiction has a significant impact on parent–child relationships.	**H1a**: The higher the child’s loss of control, the lower the intimacy, the higher the conflict, and the higher the dependency in the parent–child relationship.
	**H1b**: The stronger the child’s withdrawal, the lower the intimacy, the higher the conflict, and the higher the dependency in the parent–child relationship.
	**H1c**: The stronger the child’s escape behavior, the lower the intimacy, the higher the conflict, and the higher the dependency in the parent–child relationship.
	**H1d**: The stronger the child’s inefficiency, the lower the intimacy, the higher the conflict, and the higher the dependency in the parent–child relationship.
**Hypothesis 2 (H2)**: Smartphone addiction significantly affects children’s negative emotions and emotional regulation difficulties.	**H2a**: The stronger the child’s loss of control, the more likely they are to experience negative emotions and difficulty in emotional regulation.
	**H2b**: The stronger the child’s withdrawal, the more likely they are to experience negative emotions and difficulty in emotional regulation.
	**H2c**: The stronger the child’s escape behavior, the more likely they are to experience negative emotions and difficulty in emotional regulation.
	**H2d**: The stronger the child’s inefficiency, the more likely they are to experience negative emotions and difficulty in emotional regulation.
**Hypothesis 3 (H3)**: Children’s negative emotions and emotional regulation difficulties significantly affect parent–child relationships.	**H3**: The higher the child’s negative emotions and emotional regulation difficulties, the lower the intimacy, the higher the conflict, and the higher the dependency in the parent–child relationship.

H1a, Hypothesis 1a; H1b, Hypothesis 1b; H1c, Hypothesis 1c; H1d, Hypothesis 1d; H2a, Hypothesis 2a; H2b, Hypothesis 2b; H2c, Hypothesis 2c; H2d, Hypothesis 2d.

In conclusion, many studies have focused on smartphone addiction, negative emotions, and parent–child relationships, but research targeting elementary school students, a specific age group, is relatively limited. Elementary school children have unique characteristics in terms of cognitive, emotional, and social development, and their smartphone usage behaviors and their impact on parent–child relationships may differ from those of other age groups. Therefore, it is of great theoretical and practical significance to explore the effects of smartphone addiction and negative emotions on the parent–child relationship in elementary school children.

Additionally, based on our research, few studies have employed structural equation modeling on this topic. The objective of this study was to address this research gap through carrying out an empirical investigation on elementary school students, analyzing the impact of smartphone addiction on negative emotions and emotional regulation difficulties, as well as the effects of smartphone addiction, negative emotions, and emotional regulation difficulties on the parent–child relationship. This study employed structural equation modeling for path analysis. The results are expected to provide scientific evidence for family and school education, helping parents and educators guide elementary school children to use smartphones rationally, improve parent–child relationships, and promote children’s healthy development.

## 2 Methods

### 2.1 Participants

This study used the Mobile Phone Addiction Index (MPAI), Emotion Questionnaire, and Parent–Child Relationship Scale (CPRS) completed by parents to collect data. The study focuses on a Chinese public elementary school; participants were students aged 6–15 years; students were in grades one through six (16.78%, 18.44%, 16.70%, 16.78%, 17.05%, and 14.25%, respectively). Boys accounted for 52.88% and girls accounted for 47.12%, indicating a balanced gender distribution. The parents’ educational levels were relatively high, mostly at the undergraduate level, and their occupations were diverse. These characteristics suggest that the sample was representative and diverse, providing rich data support for this study. (**Tables 2**, **3**).

**Table 2:** Basic Indicators.

Items	N	Min	Max	M	SD	Med
Student age	1144	6.000	15.000	9.121	1.844	9.000
Parent age	1144	30.000	70.000	38.409	5.209	38.000

N, Sample size; Min, Minimum; Max, Maximum; M Mean; SD, Standard deviation; Med, Median.

**Table 3:** Descriptive Analysis.

Items	Category	Freq	Pct (%)	Cum. Pct (%)
Student Gender	M	605	52.88	52.88
	F	539	47.12	100.00
Student Grade	G 1	192	16.78	16.78
	G 2	211	18.44	35.23
	G 3	191	16.70	51.92
	G 4	192	16.78	68.71
	G 5	195	17.05	85.75
	G 6	163	14.25	100.00
Only Child	Yes	604	52.80	52.80
	No	540	47.20	100.00
Parent’s Education Level	Junior High School or Below	111	9.70	9.70
	High School	142	12.41	22.12
	Associate Degree	293	25.61	47.73
	Bachelor’s	511	44.67	92.40
	Master’s	74	6.47	98.86
	Doctorate or Above	13	1.14	100.00
Parent’s Occupation	Government/Public Administration	173	15.12	15.12
	Business Management/Senior Staff	162	14.16	29.28
	Teacher/Educator	97	8.48	37.76
	Healthcare/Medical Worker	76	6.64	44.41
	Technology/Research and Development Personnel	15	1.31	45.72
	Service Industry Worker/Manufacturing Worker	233	20.37	66.08
	Full-time Homemaker	218	19.06	85.14
	Other	170	14.86	100.00
Total		1144	100.0	100.0

Freq, Frequency; Pct (%), Percentage; Cum. Pct (%), Cumulative Percentage; M, Male; F, Female; G, Grade.

### 2.2 Measures

#### 2.2.1 Smartphone addiction

The Mobile Phone Addiction Index (MPAI), also known as the Mobile Addiction Index, is a Chinese version of the scale developed by Professor Leung Wing Chi at the Chinese University of Hong Kong. This scale was based on the diagnostic criteria for addiction from the American Psychiatric Association’s DSM-IV-TR (Diagnostic and Statistical Manual of Mental Disorders, 4th Edition, Text Revision) (51, 53). The Chinese version was created through back translation. This scale, comprising 17 items, uses a 5-point Likert scale (1 = never, 5 = always) and includes four factors: loss of control (spending excessive time on the phone without self-control), withdrawal (emotional reactions such as frustration when unable to use the phone), escapism (using the phone to escape loneliness or anxiety), and inefficiency (how excessive use affects daily life and study efficiency). The Chinese version of the MPAI has shown excellent reliability and validity in previous studies (54–57). In this study, the Cronbach’s α coefficient is 0.942, indicating high reliability, and the KMO value is 0.865, which is above 0.6, indicating good data suitability for factor analysis.

#### 2.2.2 Negative emotions and emotional regulation

The Emotion Questionnaire (58) was used to assess the emotional aspects and emotional regulation abilities of children aged 5-8. It covers the following four aspects: anger, fear, positive excitement, and sadness. Each aspect includes items on emotion (EM) and emotional regulation (ER). The emotion items measure the frequency and intensity of children’s emotional responses, whereas the regulation items assess children’s ability to regulate their emotions independently and with external assistance. This study focused on emotion items related to negative emotions (anger, fear, and sadness) and difficulties in emotional regulation of negative emotions. A 5-point scale was used; notably, a higher score on emotional regulation items indicates greater difficulty in regulating emotions. The Cronbach’s α coefficient for this measurement was 0.983, with communalities for all items above 0.4 and a KMO value of 0.922, which supported the high reliability and validity of the questionnaire.

#### 2.2.3 Parent–child relationship

The Child-Parent Relationship Scale (CPRS) developed by Pianta (52) was used to measure the quality of the parent–child relationship. The original scale included 30 items covering three dimensions: intimacy, conflict, and dependency in the parent–child relationship. A 5-point Likert scale was used. Based on theoretical analysis and a review of past research data, 13 representative items were selected to cover the core dimensions of the parent–child relationship (intimacy, conflict, and dependency), while reducing the number of questions to minimize the time and energy required from participants. The reliability and validity analysis of this measure shows good results, with a Cronbach’s α coefficient of 0.911, a KMO value of 0.834, and the variance explained by the three factors being 41.523%, 30.278%, and 23.206%, indicating that the information in the study can be effectively extracted.

### 2.3 Procedure

This study was conducted in a public primary school in China. The study participants were primary school students aged 6–15 years old. Paper-based questionnaires were randomly distributed to the parents of students. The research process was designed to ensure compliance with ethical norms, data accuracy, and the protect participants’ privacy. Prior to data collection, the study obtained ethical approval from the ethics review committee. Informed consent forms were distributed to the parents or guardians of participating students. The consent forms detailed the purpose of the study, the voluntary nature of participation, and the confidentiality of the collected data. To ensure the accuracy and integrity of data collection, the questions on the standardized questionnaires were combined into one questionnaire for distribution. In addition, demographic information including age, gender, grade, and parental education level was collected to ensure the diversity and representativeness of the sample. After the questionnaires were distributed to parents, they were required to complete them based on their observations of their children’s smartphone usage, emotional states, and parent–child interactions. In total, 1,147 questionnaires were collected, and 1,144 were valid. It needs to be specifically mentioned that this study population primarily consisted of Grade 1–6 students (n=1,143, ages 6–12 years) from an elementary school, with a single 15-year-old participant (0.09% of total sample) representing an extreme value due to delayed school enrollment. Given the certain peculiarity of the sample’s age range, when conducting data analysis, to ensure the scientific nature and accuracy of the results, the sample was divided according to the first to sixth grades of primary school.

## 3 Results

### 3.1 ANOVA results of various indicators across different grades

In this study, an analysis of variance was first performed on multiple indicators of elementary school students in different grade levels (grades 1 - 6), including dimensions related to smartphone addiction, negative emotions, emotional regulation difficulties, and various dimensions of the parent - child relationship. The results are presented as follows: (**Table 4**).

**Table 4:** ANOVA Results of Various Indicators across Different Grades.

Indicators	Student Grade: (M ± SD)						*F*	*p*
	Grade 1 (*n*=192)	Grade 2 (*n*=211)	Grade 3 (*n*=191)	Grade 4 (*n*=192)	Grade 5 (*n*=195)	Grade 6 (*n*=163)		
dependency	2.72 ± 1.05	2.75 ± 1.20	2.75 ± 1.12	2.73 ± 1.24	2.91 ± 1.32	3.24 ± 1.46	4.698	0.000**
conflict	2.30 ± 0.91	2.21 ± 0.94	2.34 ± 0.98	2.25 ± 0.87	2.40 ± 1.11	2.63 ± 1.12	4.179	0.001**
intimacy	4.02 ± 0.99	3.60 ± 1.29	3.89 ± 1.02	3.79 ± 1.15	3.82 ± 1.21	3.49 ± 1.36	4.947	0.000**
emotional regulation difficulties	2.03 ± 1.12	2.27 ± 1.31	2.17 ± 1.29	2.23 ± 1.46	2.35 ± 1.50	2.79 ± 1.67	5.971	0.000**
Negative emotions	1.82 ± 1.13	2.02 ± 1.41	2.01 ± 1.34	2.16 ± 1.49	2.25 ± 1.57	2.79 ± 1.75	9.299	0.000**
inefficacy	1.38 ± 0.58	1.48 ± 0.75	1.50 ± 0.64	1.49 ± 0.71	1.64 ± 0.89	1.74 ± 0.84	5.552	0.000**
escapism	1.46 ± 0.66	1.49 ± 0.73	1.54 ± 0.69	1.57 ± 0.75	1.56 ± 0.86	1.68 ± 0.89	1.743	0.122
withdrawal	1.35 ± 0.65	1.52 ± 0.79	1.45 ± 0.76	1.54 ± 0.80	1.69 ± 0.97	1.93 ± 0.95	10.729	0.000**
loss of control	1.66 ± 0.64	1.69 ± 0.80	1.84 ± 0.77	1.81 ± 0.70	1.77 ± 0.82	1.88 ± 0.79	2.358	0.038*

* p<0.05 ** p<0.01.

The results show that there is no significant difference in the escapism index among students in different grades (p > 0.05). However, there are significant differences (p < 0.05) among students in different grades in the eight indicators of dependency, conflict, intimacy, difficulties in emotional regulation, negative emotions, inefficiency, withdrawal, and loss of control.

Specifically, in the dimension of parent - child relationships, the scores of Grade 6 students in dependency (3.24 ± 1.46) and conflict (2.63 ± 1.12) are significantly higher than those of students in other grades. The intimacy score of Grade 1 students (4.02 ± 0.99) is significantly higher compared to students in other grades.

In the dimensions of emotional regulation difficulties and negative emotions, the scores of Grade 6 students (2.79 ± 1.67; 2.79 ± 1.75) are significantly higher.

In the dimension of smartphone addiction, Grade 6 students have significantly higher scores in inefficiency and withdrawal (1.64 ± 0.89; 1.93 ± 0.95). Grade 3 and Grade 6 students have higher scores in loss of control (1.84 ± 0.77; 1.88 ± 0.79).

### 3.2 Structural equation modeling

Based on the research hypotheses and theoretical foundation, Amos software was used to construct a structural equation model (SEM). The four factors of smartphone addiction (loss of control, withdrawal, escapism, and inefficiency) were treated as independent variables, and negative emotions and emotional regulation difficulties were treated as dependent variables to construct a path model of smartphone addiction’s effect on negative emotions and emotional regulation difficulties. Another model was constructed with smartphone addiction (loss of control, withdrawal, escapism, and inefficiency), negative emotions, and emotional regulation difficulties as independent variables and the three dimensions of parent–child relationships (intimacy, conflict, and dependency) as dependent variables to explore the effects of these factors on parent–child relationships.

**Figure 1:**
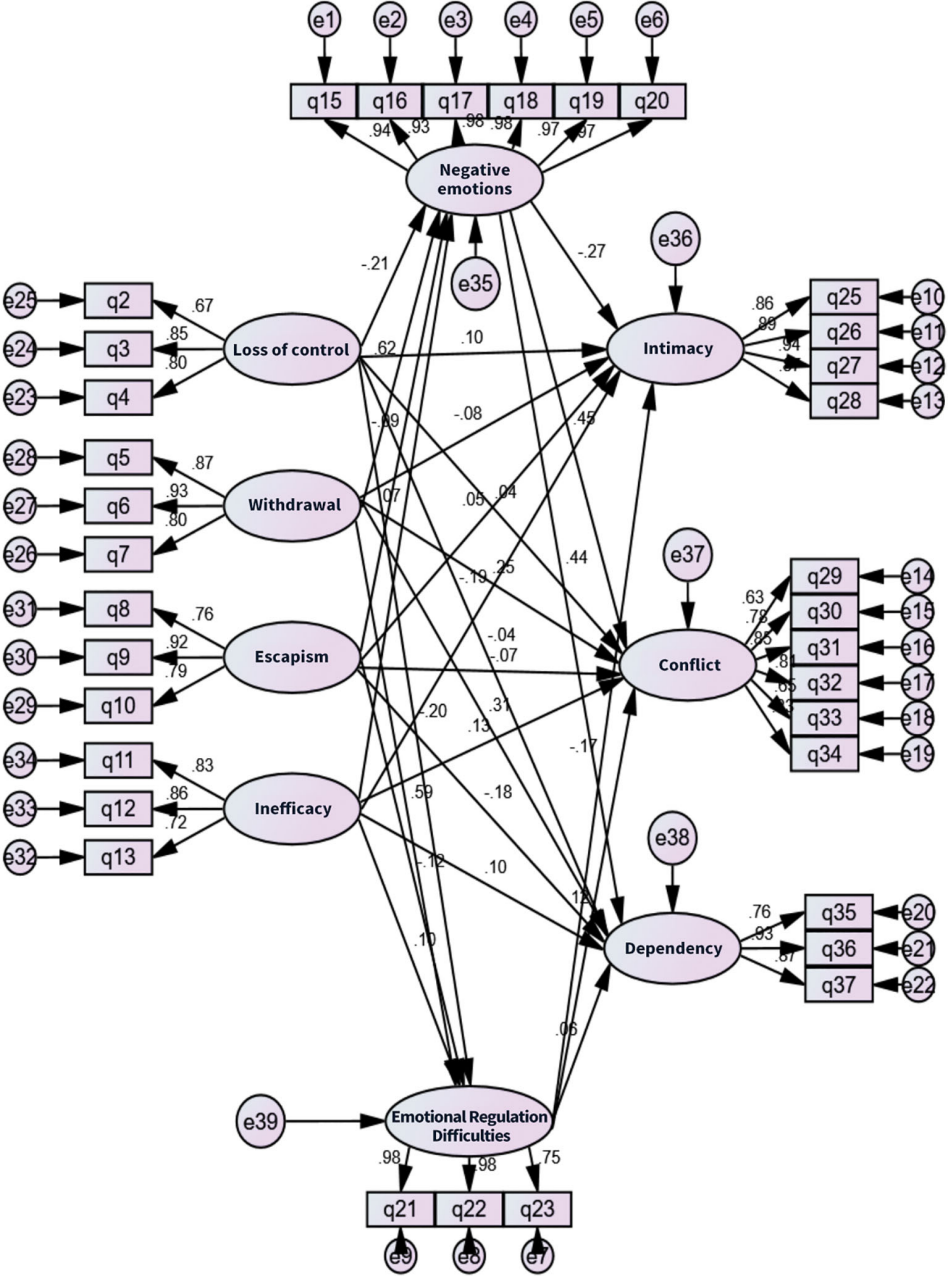
Diagram of path relationship structural equation modeling.

Subsequently, a series of fit indices was used to test the overall model fit. All fit indices (such as χ²/df, RMSEA, CFI) met the standards, indicating good model fit and suggesting the model effectively explained the variance in the data.(**Table 5**, **Figure 1**).

**Table 5:** Overall Goodness-of-Fit Index.

Statistical Test Statistic	χ2/*df*	GFI	RMSEA	RMR	CFI	NFI	NNFI
Fit Standard	<3	>0.9	<0.10	<0.05	>0.9	>0.9	>0.9
Observed Value	1.523	0.944	0.015	0.024	0.914	0.913	0.911
Model Fit	Yes	Yes	Yes	Yes	Yes	Yes	Yes

χ²/df: Chi-Square to Degrees of Freedom Ratio; GFI :Goodness-of-Fit Index; RMSEA: Root Mean Square Error of Approximation; RMR: Root Mean Square Residual; CFI: Comparative Fit Index; NFI: Normed Fit Index; NNFI: Non-Normed Fit Index.

### 3.3 Path analysis of structural equation modeling

Finally, based on the well-fitted structural equation model, path analysis was conducted to systematically explore the significance of the influence paths of the four core dimensions of children’s smartphone addiction (loss of control, withdrawal, escapism, inefficiency) on their emotional states (negative emotions, difficulties in emotion regulation). Furthermore, the direct or indirect impacts of children’s smartphone addiction and emotional states on parent-child relationships (intimacy, conflict, dependence) were explored. Key findings revealed that (**Tables 6**, **7**):

**Table 6:** Model Path Coefficient Fit Summary.

X	Path	Y	Est.	S.E.	Z (C.R.)	P
loss of control	→	negative emotions	-0.213	0.054	-8.257	***
loss of control	→	emotional regulation difficulties	-0.197	0.05	-7.325	***
loss of control	→	intimacy	0.101	0.051	3.19	**
loss of control	→	conflict	0.037	0.031	1.357	n.s.
loss of control	→	dependency	-0.043	0.04	-1.691	n.s.
withdrawal	→	negative emotions	0.621	0.055	21.982	***
withdrawal	→	emotional regulation difficulties	0.587	0.055	18.557	***
withdrawal	→	intimacy	-0.076	0.067	-1.715	n.s.
withdrawal	→	conflict	0.247	0.042	6.267	***
withdrawal	→	dependency	0.314	0.055	8.406	***
escapism	→	negative emotions	-0.085	0.06	-3.491	***
escapism	→	emotional regulation difficulties	-0.119	0.055	-4.656	***
escapism	→	intimacy	0.05	0.054	1.763	n.s.
escapism	→	conflict	-0.071	0.033	-2.871	**
escapism	→	dependency	-0.177	0.044	-7.417	***
inefficacy	→	emotional regulation difficulties	0.101	0.053	3.886	***
inefficacy	→	negative emotions	0.071	0.057	2.853	**
inefficacy	→	dependency	0.098	0.041	4.146	***
inefficacy	→	conflict	0.129	0.032	5.06	***
inefficacy	→	intimacy	-0.187	0.052	-6.312	***
Negative emotions	→	conflict	0.451	0.02	12.436	***
Negative emotions	→	intimacy	-0.267	0.029	-7.097	***
Negative emotions	→	dependency	0.44	0.024	13.576	***
emotional regulation difficulties	→	dependency	0.06	0.025	2.063	*
emotional regulation difficulties	→	conflict	0.119	0.019	3.812	***
emotional regulation difficulties	→	intimacy	-0.166	0.032	-4.545	***

X, Independent Variable; Y, Dependent Variable; Est., Estimate; S.E., Standard Error; Z (C.R.), Z-value (Critical Ratio); P, P value; n.s., nonsignificant; *p<.05; **p<.01:***p<.001.

**Table 7:** Results.

**R1a**: The higher the child’s loss of control, the higher the intimacy, with no significant impact on conflict and dependence in the parent–child relationship.
**R1b**:The stronger the child’s withdrawal, the higher the conflict and dependence, with no significant impact on intimacy in the parent–child relationship.
**R1c**: The stronger the child’s escape behavior, the lower the conflict and dependence, with no significant impact on intimacy in the parent–child relationship.
**R1d**: The stronger the child’s inefficiency, the lower the intimacy, the higher the conflict, and the higher the dependency in the parent–child relationship.
**R2a**:The stronger the child’s loss of control, the less likely they are to experience negative emotions and easy in emotional regulation.
**R2b**: The stronger the child’s withdrawal, the more likely they are to experience negative emotions and difficulty in emotional regulation.
**R2c**: The stronger the child’s escape behavior, the less likely they are to experience negative emotions and easy in emotional regulation.
**R2d**: The stronger the child’s inefficiency, the more likely they are to experience negative emotions and difficulty in emotional regulation.
**R3**: The higher the child’s negative emotions and emotional regulation difficulties, the lower the intimacy, the higher the conflict, and the higher the dependency in the parent–child relationship.

R1a, Result 1a; R1b, Result 1b; R1c, Result 1c; R1d, Result 1d; R2a, Result 2a; R2b, Result 2b; R2c, Result 2c; R2d, Result 2d; R3, Result 3.

• Smartphone addiction dimensions divergently impacted emotions: Withdrawal (intense anxiety/irritability upon smartphone deprivation) and inefficiency (impaired functioning due to excessive use) demonstrated robust emotional deterioration effects, positively predicting negative emotions (β = 0.621, p < 0.001;β = 0.071, p = 0.004) and emotion dysregulation (β = 0.587, p < 0.001;β = 0.101, p < 0.001). While loss of control and escapism (device-mediated avoidance of real-world stressors) exerted inhibitory effects on internal negative emotions (β = -0.213, p < 0.001;β = -0.085, p < 0.001) and emotion regulation difficulties (β = -0.197, p < 0.001;β = -0.119, p < 0.001), suggesting that when children use smartphones to escape from real-life pressures and lose control of their smartphone use, it can, to a certain extent, reduce children’s emotional burden.

These findings challenge the linear assumption in traditional family systems theory that “loss of control inevitably degrades relationships,” which primarily derives from adult usage patterns under parental supervision. In addition, this study also reminds us to be wary of its potential risks — if children rely on mobile phones as an emotional regulation tool for a long time, it may cover up their real needs and lead to more profound behavioral problems.

• Differential effects of smartphone addiction on parent - child relationships: Loss of control positively predicted parent-child closeness (β = 0.101, p = 0.001) while showing no significant effects on conflict (β = 0.037, p = 0.175) or dependency (β = -0.043, p = 0.091). This implies that uncontrolled smartphone use may transiently enhance parent-child bonding by reducing emotional burden. Withdrawal exacerbated parent-child conflict (β = 0.247, p < 0.001) and dependency (β = 0.341, p < 0.001) without affecting closeness (β = -0.076, p = 0.086). These patterns indicate that withdrawal behaviors fuel relational dysfunction, manifesting as confrontational resistance to device restriction or manipulative compliance-seeking tactics. Escapism reduced conflict (β = -0.071, p = 0.004) and dependence (β = -0.177, p < 0.001), though failed to enhance closeness (β = 0.05, p = 0.078).This finding suggests that escaping from reality may alleviate the parent-child relationship in the short term, but it fails to effectively promote positive emotional bonds. Inefficiency led to high conflict (β = 0.129, p < 0.001), high dependence (β = 0.098, p < 0.001), and low intimacy (β = -0.187, p < 0.001).This confirms that inefficient behavior leads to an individual’s internal emotional burden and also seriously affects the quality of parent-child relationships.

• The impact of emotions caused by smartphone addiction on parent - child relationships: Notably, negative emotions and emotion regulation difficulties significantly mediated smartphone addiction’s impact on relational outcomes: positively predicting conflict(β = 0.451, p < 0.001;β = 0.119, p < 0.001) and dependency (β = 0.44, p < 0.001;β = 0.06, p = 0.039), while negatively influencing closeness (β = -0.267, p < 0.001;β = -0.166, p < 0.001). This result indirectly indicates that children’s internal emotional state is a crucial bridge through which smartphone addiction affects parent-child relationships.

The research results suggest that when smartphone use becomes the main means of children’s emotional regulation (such as uncontrolled mobile phone use, escapism), we need to be particularly vigilant about its potential risks. Therefore, it is suggested that parents should rationally view the length of time their children use mobile phones, allow moderate use to relieve emotions under supervision, but set clear boundaries to prevent it from becoming a tool for escaping reality. Similarly, the high destructiveness of smartphone withdrawal indicates that a simple and rough ban strategy may exacerbate parent - child conflicts. Parents need to go beyond the stereotype that “addiction is harmful,” deeply analyze the specific motivations of children’s mobile phone use (such as stress relief, social needs), and through empathetic communication and cooperation, formulate usage rules to protect the parent - child relationship while guiding children to develop self - discipline.

## 4 Discussion

This study investigated the impact of smartphone addiction on elementary school students’ negative emotions, emotional regulation difficulties, and their subsequent effects on parent–child relationships. The research results are of great significance to parents and educators, and can help promote children’s healthy growth and harmonious parent - child relationships.

When exploring the complex relationships among smartphone addiction, negative emotions, and parent - child relationships, the grade differences among students are important factors that cannot be ignored. Elementary school students in different grades have obvious differences in cognitive development, psychological maturity, and living environments. These differences are likely to have varying degrees of impact on their behavioral patterns of smartphone use, emotional states, and the quality of parent - child relationships. In order to reveal these potential differences, this study first used the method of analysis of variance to systematically analyze the data of elementary school students in different grades on various indicators.

Judging from the results of the analysis of variance, students in all grades show relatively consistent degrees of using smartphones to escape real - world problems. However, there are significant differences among students in different grades in the eight indicators of dependency, conflict, intimacy, difficulties in emotional regulation, negative emotions, inefficiency, withdrawal, and loss of control.

In terms of parent - child relationships, Grade 6 students are more dependent on their parents. This may be because as the grade level increases, the complexity of learning and life also increases. When facing difficulties, Grade 6 students rely more on their parents. At the same time, the self - awareness of students in this grade has increased, and it is easier for them to have differences in opinions with their parents, thus triggering conflicts. The parent - child intimacy of Grade 1 students is higher because lower - grade students have a stronger emotional dependence on their parents. As students grow older and their independence develops, their intimacy with parents decreases. In terms of difficulties in emotional regulation and negative emotions, Grade 6 students have higher scores. This is related to the fact that senior - grade students face more academic pressure and complex interpersonal relationships, resulting in more complex emotional experiences. In terms of smartphone addiction, Grade 6 students have significantly higher scores in the dimension of inefficiency. This may be because senior - grade students have heavy learning tasks, and smartphone use has a greater impact on their study and life. At the same time, students in this grade have a higher degree of dependence on smartphones and more intense withdrawal reactions. In terms of loss of control, the scores of Grade 3 and Grade 6 students are generally higher. This is because Grade 3 and Grade 6 are crucial stages in primary - school learning. The change in pressure may cause students to be more likely to lose control over their smartphone use.

In the exploration of smartphone addiction among primary school students, apart from the differences in the degree of addiction among children in different grades, there are also fundamental differences between children’s and adults’ smartphone addiction. The core of these differences stems from the characteristics of the developmental stage and insufficient socialization levels. Compared with adults, children lack mature social constraints and a sense of responsibility, and their self-regulation ability has not been fully formed (59). For example, adults may take the initiative to manage their smartphone usage time due to professional needs or social obligations, while children’s smartphone usage behavior is more driven by immediate emotions (60) (such as avoiding academic pressure or seeking entertainment and excitement), and they lack foresight about the long-term consequences of their behavior (such as a decline in learning efficiency and social alienation) (61).In addition, the dominance of family supervision is an important context for children’s addiction: Adults usually rely on an internal sense of responsibility for self-restraint (62), while the boundaries of children’s smartphone usage are mainly set by their parents. This external dependence leads to unique behavioral patterns in children in terms of loss of control (using the phone overtime even under supervision) and withdrawal symptoms (more intense emotional reactions). For example, children may repeatedly challenge the rules because they cannot understand the significance of “usage time limits,” or they may exhibit more extreme anxiety reactions (such as crying and aggressive behavior) after their phones are confiscated. The neural basis of this difference is also worthy of attention: The prefrontal cortex of children (which is responsible for executive control and decision-making) is not yet fully developed, making them more vulnerable to the drive of immediate rewards (such as game achievements and social media likes) and difficult to suppress impulsive usage (63). In contrast, the prefrontal function of adults is more complete, and they can regulate their behavior through rational weighing (such as “using the phone will affect work efficiency”).

Therefore, the interpretation of the outcomes of children’s smartphone addiction and subsequent interventions need to take into account the uniqueness of their cognitive and social development, rather than simply referring to the relevant research results and intervention strategies for adults.

A path analysis of the effects of smartphone addiction on negative emotions and emotional regulation revealed that when children use smartphones as a means of escaping stress (escape behavior refers to using smartphones to avoid real-life problems, emotional distress, or stress) or when they experience uncontrollable smartphone usage (where individuals struggle to control the time or frequency of smartphone use), negative emotions may be alleviated, and emotional regulation may become easier.

Preliminary research has indicated that many adolescents with Internet addiction display more problematic Internet use behaviors that are linked to greater negative emotions and more severe emotional regulation difficulties (40). Additionally, smartphone addiction exacerbates negative emotions and difficulties in emotional regulation (6, 14, 41). However, this study provided new and innovative evidence that moderate smartphone use may, to some extent, suppress negative emotions and help improve children’s emotional regulation difficulties. Two theoretical perspectives help explain this mechanism: the compensatory internet use theory (CIUT) and emotional information theory.

- CIUT suggests that smartphones are often used to relieve negative emotions and meet social needs (34). Children can seek comfort and satisfaction in the virtual world, temporarily escaping real-life stress and distress, which, in turn, helps suppress negative emotions.
- The emotional information theory posits that emotions are determined by the gap between the information an individual has and the information they need. When available information is insufficient, negative emotions arise (64). In this context, smartphones and similar virtual resources may serve as substitutes to fill the information gap (65). Moreover, smartphone apps often provide immediate feedback and rewards, enhancing children’s positive emotional experiences (66), which help alleviate negative emotions and improve emotional regulation difficulties.

These theories suggest that negative emotions and difficulties in emotional regulation often trigger smartphone use and addiction, with excessive use becoming a compensatory behavior to alleviate negative emotions (67). Over time, this compensatory behavior is reinforced by a reduction in negative emotions (68), leading to further smartphone use and the development of a vicious cycle. Emotional regulation enables children to avoid overwhelming negative emotions (69). However, excessive smartphone use as a compensatory behavior causes only short-term emotional numbness and does not lead to emotional healing. Children repeatedly experience brief moments of happiness from this easily accessible compensation. Their ability to self-regulate emotions becomes weaker over time. This discovery serves as a warning that when smartphones become the primary means of emotional regulation for children, the potential risks cannot be ignored. Therefore, it is recommended that parents rationally view the length of time their children use smartphones. Under the premise of supervision, they should allow moderate use to relieve emotions. However, at the same time, clear boundaries must be set to prevent smartphones from becoming a tool for children to escape from reality.

In addition, the study showed that withdrawal symptoms (i.e., anxiety, discomfort, irritability, or other unpleasant feelings when a smartphone is not available) and inefficiency (i.e., the impact of excessive smartphone use on normal life, learning, or work efficiency) exacerbate negative emotions and further hinder emotional regulation. Smartphone addiction creates dependency. When use is halted, withdrawal reactions similar to substance addiction occur (70), leading to negative emotions. Stopping smartphone use disrupts habitual behavioral patterns, and children feel anxious when they do not use their phones (71, 72). According to the CIUT, excessive smartphone use may serve to escape negative emotions (34); however, once smartphone use is stopped, children can no longer escape and are forced to face and directly process their negative emotions. As elementary school students often lack emotional regulation strategies (73), the absence of a smartphone as an external emotional regulation aid makes emotional regulation even more difficult. Additionally, inefficient smartphone use disrupts students’ normal lives and studies, leading to anxiety and depression (74). This may increase students’ reliance on their phones for emotional regulation, and smartphone-related information overload increases their cognitive load (75), which interferes with the emotion regulation process. This suggests a need for family education strategies to help students improve their emotion regulation abilities and effectively manage withdrawal periods to avoid withdrawal reactions and enhance their learning efficiency.

Research has found that smartphone addiction can also have an adverse impact on parent-child relationships. Specifically, loss of control smartphone use can increase parent-child intimacy. This is because the relatively lenient parental control over children’s smartphone use allows children to obtain a certain degree of emotional comfort from smartphones, which they perceive as a form of emotional support from their parents to some extent (76). Moreover, the reduced parental control over children’s smartphone use has led to fewer parent - child conflicts. Consequently, this has further contributed to the enhancement of parent - child intimacy. Simultaneously, children depend psychologically on their parents’ emotional support (24), which strengthens their emotional bonds. Withdrawal also significantly affects parent–child relationships, although it has rarely been studied systematically. The discomfort children experience when withdrawing from smartphones leads to the return of negative emotions, reducing parent–child interaction and potentially increasing conflict, which decreases intimacy (29). However, when a smartphone is removed as a compensatory object to alleviate negative emotions (26), children unconsciously rely on their parents to relieve their discomfort (24), thereby increasing dependency. The high level of disruption caused by smartphone withdrawal indicates that adopting a simple and crude strategy of banning smartphone use may exacerbate parent-child conflicts. Parents need to correct the stereotype that “addiction is always harmful”, fully understand the specific motivations behind their children’s smartphone use (whether it’s to relieve stress or meet social needs), and then help their children smoothly transition through the withdrawal period through empathetic communication and collaboration.

Escape behavior, in which children use smartphones to avoid stress, helps them separate themselves from conflictual situations (e.g., parent–child conflict), reducing opportunities for conflict with others. Avoidance is a psychological coping mechanism that reduces one’s emotional dependence on others (77). Therefore, escape behavior not only helps alleviate negative emotions (34), but also reduces emotional dependence on parents by offering temporary solace through smartphones, thus lowering the need for parental support and reducing dependency while potentially creating emotional distance (78).

Finally, inefficiency increases both emotional dependency and conflict in parent–child relationships, thereby reducing intimacy. Studies have shown that excessive digital media involvement leads to task delays and avoidance of responsibility (79). Children’s cognitive abilities, including the ability to understand and regulate emotions, are still developing (80). Therefore, parents need to provide support in managing smartphone-related issues (24), which strengthens emotional dependence and deepens parent–child bonds. On the other hand, excessive smartphone use can lead to severe parent–child conflict, which aligns with prior research. According to the Social Substitution Hypothesis, smartphone addiction uses time that would otherwise be spent on other aspects of life (81, 82). The resulting inefficiency in daily life and academic studies leads to dissatisfaction and criticism from family members, thereby increasing the likelihood and level of conflict. Moreover, excessive smartphone use reduces interaction and communication with others (83), weakens emotional connections, and lowers intimacy.

Regarding the influence of negative emotions and emotional regulation difficulties on parent–child relationships, previous studies have identified their crucial role in shaping these relationships (30, 84). However, most studies have focused on how tense parent–child relationships exacerbate children’s negative emotions (85), which may drive children further into compensatory smartphone addiction (86). In this study, negative emotions led to lower intimacy, higher conflict, and greater dependence on parent–child relationships. Frequent negative emotions in children seriously weaken parent–child intimacy. Adolescents, especially elementary school students, are less aware of the risks of using the Internet (87), and children with severe smartphone addiction tend to exhibit more significant behavioral and emotional problems (88), such as anxiety and anger, which may lead to resistance and reduced emotional sharing with parents, thereby distancing the parent–child relationship (89). Negative emotions stem from various sources, and during childhood, children seek recognition and support from their parents, peers, and other members of their social groups (90). When these needs are unmet, feelings of loneliness and other negative emotions arise, which increase the likelihood of smartphone addiction (90, 91). Accumulating negative emotions also trigger conflicts and contradictions in parent–child relationships. When children are in a negative emotional state, they may show impatience or resistance to parental discipline, escalating conflicts; this finding aligns with the existing literature (92). Negative emotions also have a significant positive impact on increasing parent–child dependency. Parents are often viewed as crucial sources of emotional support (93). Children who experience negative emotions tend to become more emotionally dependent on their parents. For instance, anxiety and anger can lead to psychological problems (94), such as unease and fear, prompting children to seek comfort and support from their parents, which enhances emotional dependence. According to attachment theory, insecure parent–child attachment reduces adolescents’ emotional warmth, social support, and sense of security (95), which exacerbates negative emotions and increases reliance on other forms of support (96). If parents do not provide timely attention and support, smartphones may become compensatory objects of emotional attachment (26).

Furthermore, emotional regulation plays an important role in parent–child relationships (97). Difficulties in emotional regulation may lead children to display unstable emotions, irritability, and other behaviors when interacting with parents, weakening parent–child intimacy. Uncontrolled emotions can also lead to arguments and conflicts that exacerbate tensions in relationships. These findings are consistent with those of previous studies, particularly regarding dependency. Children may become more reliant on their parental emotional support to cope with emotional distress (24), leading to greater dependency. Parents play an essential part in supporting children to build up emotion - regulation techniques (98), and the empathy they possess is positively related to the attachment security and emotional openness of children (99). It can be seen that children’s internal emotional states serve as a crucial bridge connecting children’s smartphone addiction and parent - child relationships. In order to enhance the parent - child relationship, parents need to adopt appropriate intervention strategies to help their children successfully develop their self - emotional regulation abilities.

### 4.1 Limitations and options for future research

This study had some limitations. First, the cross–sectional design restricts the causal inferences made over time. Unlike longitudinal studies, this study does not clearly demonstrate the temporal effects of these variables. Additionally, the transition from childhood to adolescence is a critical period for identity development, during which children gradually gain independence from their parents, and the parent–child relationship may exhibit specific features. Furthermore, the study relied on self-reported measures, which may have introduced recall and social desirability biases. However, the analysis results of this experiment provide a new perspective and evidence for our in - depth understanding of the complex impact relationship among smartphone addiction, primary school students’ emotional states, and parent - child relationships. It also reminds parents to carry out interventions based on a thorough understanding of the impact mechanism of children’s smartphone addiction. Follow - up studies will continue to focus on the supplementary discussion of the correlation between parents’ intervention methods and the above - mentioned results. For example, exploring how parents can provide targeted guidance based on their children’s negative emotional expressions during smartphone withdrawal, and how to offer understanding and strategic support when children face negative emotions and in the process of developing their emotional regulation abilities, so as to provide more comprehensive and effective strategies for solving the problem of children’s smartphone addiction. In addition, this study was only carried out in one primary school, the generalizability of its results is limited and may not be applicable to other regions. In the subsequent in-depth research, factors such as cultural adaptability and culturally responsive evaluation in a multicultural context should be fully considered, so as to ensure that the research results and subsequent support strategies can be applied to school and family based support strategies and solutions for children’s smartphone addiction in different countries and regions (100). Given that the future research needs to conduct large - sample surveys in different regions to test and expand on the findings of this study.

## 5 Conclusion

This study explored the intricate relationships among smartphone addiction, negative emotions, and parent-child relationships in elementary school students. By employing methodologies such as structural equation modeling (SEM) and analysis of variance (ANOVA), the findings revealed critical insights: distinct dimensions of smartphone addiction (loss of control, withdrawal, escapism, and inefficiency) exerted differential impacts on emotions and parent-child relationships. Specifically, withdrawal and inefficiency exacerbated negative emotions and emotional regulation difficulties, while loss of control and escapism partially alleviated these challenges. Furthermore, both smartphone addiction and negative emotions significantly influenced parent-child relationships, manifesting as diminished intimacy, intensified conflict, and heightened dependency. Notably, senior-grade students exhibited heightened levels of addiction, negative emotions, and parent-child conflict, likely attributable to academic pressures and complex social dynamics. The study emphasizes the necessity for parents and caregivers to adopt a rational perspective on children’s smartphone use motivations, avoid simplistic prohibitions, and establish reasonable boundaries through empathetic communication. Concurrently, fostering children’s emotional regulation skills is imperative. Conducted in an era where smartphones have become an inseparable part of daily life, this research provides a scientific basis for educators and families to promote children’s healthy development and foster harmonious parent-child relationships.

## Data Availability

The original contributions presented in the study are included in the article/supplementary material. Further inquiries can be directed to the corresponding author.
